# Expanded methods for geometric location of 2D bone histology co-registration in 3D radiology volumes

**DOI:** 10.1007/s13246-026-01764-w

**Published:** 2026-07-27

**Authors:** Jaime Uy De Baron, Robert Phillips, Anthony Butler

**Affiliations:** 1 Pathology and Biological Sciences, The University of Otago, Canterbury, New Zealand; 2 Mechatronics Engineering, The University of Canterbury, Christchurch, New Zealand; 3 Anatomic Pathology, Te Whatu Ora, Canterbury, New Zealand

**Keywords:** Radiopathology, Patient specific treatment, Histology localisation, Orthopedic oncology, Geometric location

## Abstract

Bone sarcoma treatment employs wide surgical margins to ensure complete tumour excision, sometimes at the expense of patient quality of life. As precision technologies advance, quantitative data to inform the planned accuracy of surgical margins is increasingly important. This study assesses two novel computational methods (M2, M3) to support the co-registration of 2D histologic images within 3D radiological volumes. Replicated evaluation of an established method (M1) is also performed with new data. M2 suits concave bisected sections by assuming a different orientation during tissue bandsaw than M1. M3 handles freeform geometries by using a third measurement to constrain 2D histology images within 3D space without need for geometric bandsaw assumptions. Three computed distances across 38 canine specimen dissections from each method were compared with three corresponding physical measurements collected during laboratory processing. M1 produced a mean error of 0.12 (± 1.7) mm, M2 produced a mean error of 0.06 (± 1.9) mm, while M3 produced a mean error of 0.02 (± 1.5) mm. Mean errors for each method were of similar magnitude to 3D registration error reported in histology co-registration frameworks for other tissues. This study supports a simple low cost opportunity for use of digital histology images to support bone sarcoma radiology interpretation and treatment planning.

## Introduction

Bone sarcoma is rare in the general population (0.2%) but accounts for 5% of all childhood cancers [1]. In Australia, bone and soft tissue sarcomas make up just 1.5% of new cancer diagnoses in those aged 0–19, yet they are the second leading cause of cancer-related death in this group [2, 3].

This low incidence creates significant challenges in data availability, limiting the evaluation of treatment technologies that rely on pre-surgical radiological tumour interpretation, characterisation, and segmentation [4–7]. Current histological correlations provide limited conclusions for surgical guidance, typically consisting of singular classification points such as Musculoskeletal Tumour Society (MSTS) Residual (R) data [8, 9].

Aligning 2D histology within 3D volumes (i.e. co-registration) provides feature-rich spatial context to inform bone sarcoma treatment/interventions. Co-registration could improve diagnostic sensitivity, accuracy, and treatment response prediction by orders of magnitude compared to 2D pathology [10]. However, limitations of current bone sarcoma histology orientation techniques for evaluating anatomic models (such as manual 3D graphical slicing [6]) stem from poorly defined characterisation of each histology section in spatial coordinates. This may impact histology orientation accuracy and fundamentally limits the quantity of quantitative assessments possible per specimen. More objective geometric dissection plane modelling is needed to determine the spatial position of 2D histology images within 3D bone co-registration frameworks.

**Fig. 1:**
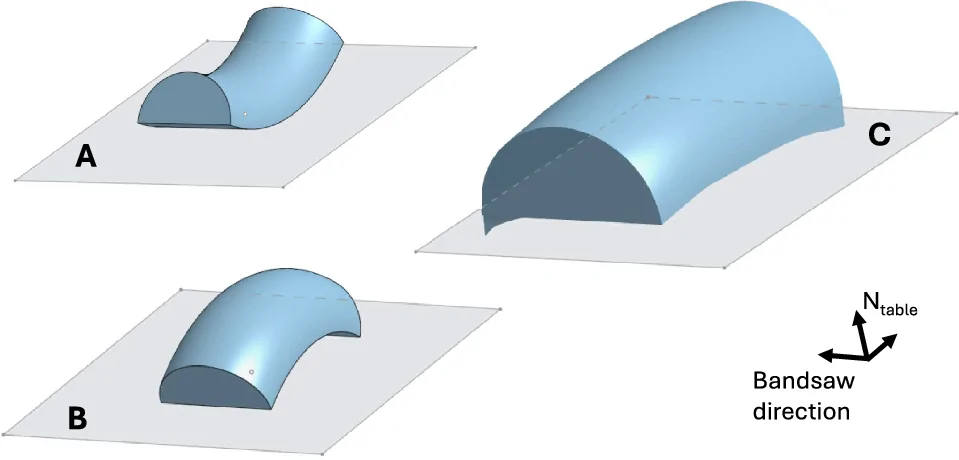
Demonstration of how different bisected bone halves may sit on a bandsaw table for dissection: **A** - Convex, **B** - Concave, **C** - More freeform. Surface **A** is conceptually suitable for the perpendicularity assumption in M1, where the dissection plane is perpendicular to the surface at the point of dissection. However, this does not apply to surfaces **B** or **C**. $N_{table}$ is the normal vector to bandsaw table, and the bandsaw direction for dissections. Images generated using *Onshape*

This study presents two novel methods for 2D dissection plane modelling in 3D to address geometric assumption limitations of an existing bone sarcoma co-registration solution [11], hereafter referred to as Method 1 (M1). M1 is an approach for assigning histology section planes within 3D bone radiology volumes based on geometric assumptions about the bisection surface and bandsaw orientation. The proposed methods accommodate irregular and concave bone geometries that determine variable bisection surfaces (see Fig. 1). The co-registration of bone histology has limitations that custom cutting jigs or internal fiducials used in prostate and other tissue [12] cannot reliably overcome [13]. The objectives of this study are to evaluate methodological advances and expand the specimen cohort from previous trials to strengthen validation of the only known current co-registration framework for clinical bone sarcoma.

### Motivation

Current bone sarcoma treatments include chemotherapy, radiation therapy, and surgical resection. However, surgery remains the primary treatment modality due to the inherent radioresistance of bone sarcomas [14], with certain subtypes such as chondrosarcoma demonstrating persistent resistance to both chemotherapy and radiotherapy [14, 15].

Surgical resection typically removes a ‘wide’ margin of 10 to 20 mm to reduce the likelihood of residual tumour cells [16, 17]. Margin status directly correlates with patient outcomes: positive surgical margins (defined by tumour cells at the edge or resected tissue) suggest poor overall survival, whereas negative margins significantly improve survival prospects [17, 18]. However healthy tissue margins inherently necessitate damage to healthy tissue, which can include nerves and blood vessels. This impairs post-surgical mobility and bodily function [19] creating a fundamental clinical dilemma: preserve healthy tissue and risks local recurrence or metastasis, or plan wider margins and compromise patient comfort and quality of life [19].

Tumours in complex areas such as the pelvis are particularly challenging [20]. Mortality rates remain high across all age groups, with a 5-year survival rate of about 65% for complex treatment of Ewing’s sarcoma and osteosarcoma [1]. These surgeries can significantly impact patient mobility and quality of life [19].

Bone sarcomas exhibit a bimodal age distribution, with particularly high incidence in younger patients [21]. The societal impact is amplified by the young age at diagnosis which premature mortality or long-term mobility impairments contribute to.

The lack of clarity on how ‘wide’ a resection should be has led healthcare clinicians and industry professionals to call for re-evaluation of current bone sarcoma treatment [7]. In particular, it has been recommended that guidelines for surgical resection margins [7] be revised. Comprehensive data is needed to balance effective tumour removal with the preservation of as much normal tissue as possible while maintaining appropriate function and mobility. A critical evaluation is urgently needed to inform modern treatment and limb salvage practices [7].

### Existing literature

Histology to radiology co-registration solutions - which include fiducial strands with manual image feature identification [22], specimen masking with software-based visualisation [23], and particle filtering [24] - have been developed for prostate tissue. These face different histology processing challenges to bone. Phillips et al. present the only clinically appropriate approach for bone tissue currently known; however, their solution employs a modelling method (M1) that assumes convex or perpendicular dissection planes [11]. This approach may have reduced accuracy when applied to concave or complex bisection surfaces.

We introduce two new methods (M2 and M3) that build on this published bone sarcoma framework [11]. Our methods are designed to handle concave and more freeform bone geometries.

## Method

### Specimen selection, preparation, and imaging

Four canine tissue specimens were sourced from Massey University based on tissue availability, with prior owner consent. A reference hole was added to each specimen using a drill press. Then they were longitudinally bisected into approximate halves, where bone geometry permitted, with a diamond bandsaw before ex-vivo CT imaging.

High-definition CT imaging was performed using a Revolution GSI scanner (GE Medical Systems, USA) at Lincoln, New Zealand. The protocol utilised 0.625 mm slice axial spatial reconstruction with 120 kVp, 0.5 pitch, and 512 x 512 matrix parameters. All processing was conducted on fresh tissue in accordance with pathology protocols [25] as freezing may cause ice crystal formation that compromises clinical diagnostic accuracy.

### Definitions

**Fig. 2:**
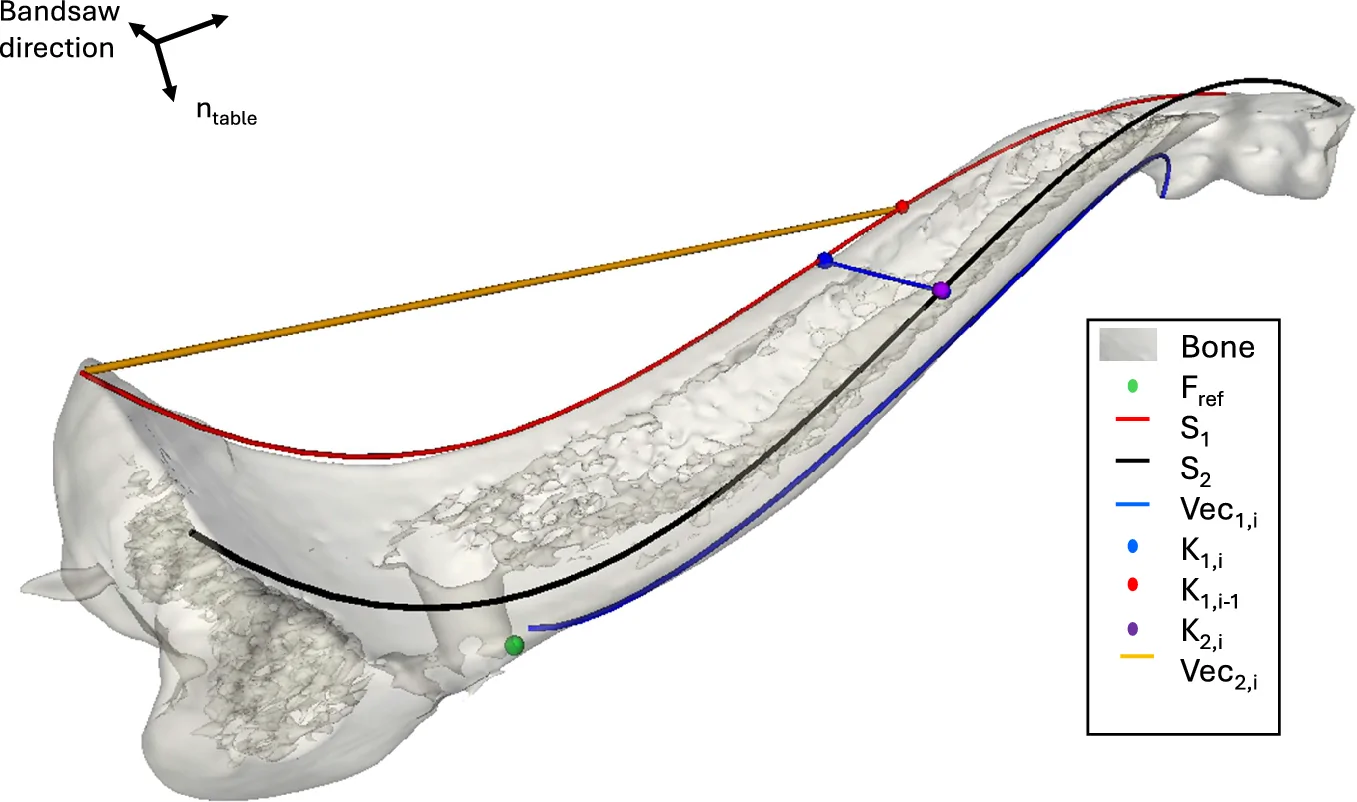
$\boldsymbol{Vec_{1}}$ and $\boldsymbol{Vec_{2}}$ displayed on the two mark-up splines along the bisection surface, 3D Python diagram render using trimesh *(a Python-based 3D geometry processing library)*

A list of all variables used in the M2 procedure is included below and demonstrated in Fig. 2.

- $F_{ref}$ - Fiducial reference point, marked on *3D Slicer*, at the position of the physical reference hole
- $S_{1}, S_{2}$ - Splines 1 and 2, marked up on *3D Slicer*, used for primary calculations
- $P_{n}$ - An intersection point as determined by Python, *the point on the spline* that exists on the cut plane
- $\boldsymbol{Vec_{1}}$ - The vector between two paired intersection points on each of the two splines
- $\boldsymbol{Vec_{2}}$ - The vector between the endpoint of a spline to the intersection point of the most recent cut made
- $N_{1}$ - The vector normal to the plane spanned by $\boldsymbol{Vec_{1}}$ and $\boldsymbol{Vec_{2}}$
- $N_{2}$ - The vector normal to the plane spanned by $N_{1}$ and $\boldsymbol{Vec_{1}}$
- $K_{1,i}$ - The intersection point of $i^{th}$ Euclidean measurement and $S_{1}$ defined in x, y, z coordinates
- $K_{2,i}$ - The Python-determined intersection point of $i^{th}$ Euclidean measurement and $S_{2}$ defined in x, y, z coordinates
- $K_{1,i-1}$ - The Python-determined intersection point of the previous Euclidean measurement and $S_{1}$ defined in x, y, z coordinates

### Method 2 Computational procedure

#### Physical processing

Bisections were dissected following CT with the previously created bisected surface resting on the table. After each planar bandsaw dissection, a measurement was taken (with vernier callipers) from the fiducial reference point to the two furthest corners of the newest cut face.

#### Step 1 - Spline mark-up

The CT volume was input into *3D Slicer* *(an open-source medical image visualisation and analysis platform)* and two splines were marked up that ran along the edges of the bisection surface. $F_{ref}$ was also placed approximately at the location of the drill hole. These 3 geometries were exported as.json files for Python processing.

#### Step 2 - Spline calculations

Using the spline mark-up points, a $3^{rd}$ order polynomial was fitted in *Python 3.12* *(a programming language used here for numerical computation)* using polyfit, a function that finds the least squares fit to a set of points [26]. A cubic polynomial was deemed suitable, as bone structures rarely follow the shape of higher order curves [27]. The Euclidean measurements from the fiducial to the intersection point were utilised to determine the points of each cut plane intersection ($K_{1,i}$ and $K_{2,i}$) using fsolve, a function that uses the Newton–Raphson method to find the roots of an equation [28]. Intersection points on one spline (e.g. $K_{2,i}$) could then be subtracted from those on the corresponding spline (e.g. $K_{1,i}$) to obtain $\boldsymbol{Vec_{1}}$.

#### Step 3 - Normal to plane calculations

The vector between the most recent cut plane and the fiducial side spline endpoint $\boldsymbol{Vec_{2}}$ was calculated. The cross product of $\boldsymbol{Vec_{1}}$ and $\boldsymbol{Vec_{2}}$ formed $N_{1}$. The cross product of $\boldsymbol{Vec_{1}}$ and $N_{1}$ forms $N_{2}$, the normal of the plane of the perceived cut surface.

#### Step 4 - Plane coefficients

The dot product of $N_{2}$ and $K_{2,i}$ gave the constant term for the plane equation. Thus, the plane equation in the form *ax + by + cz = d* was produced. These coefficients were written into a.json file to export back into *3D Slicer* for histology image assignment.

### Method 3 Computational procedure

#### Additional definitions

Additional variables used in the M3 procedure are included below.

- $S_{3}$ - Spline 3, marked up on *3D Slicer*, used for calculation
- $\boldsymbol{Vec_{3}}$- The vector between intersection points on $S_{1}$ and $S_{3}$
- $\boldsymbol{Vec_{4}}$ - The vector between intersection points on Spline 2 and Spline 3
- $N_{3}$ - The vector normal to the plane spanned by $\boldsymbol{Vec_{1}}$ and $\boldsymbol{Vec_{2}}$
- $K_{3,i}$ - The Python-determined intersection point of $i^{th}$ Euclidean measurement and $S_{3}$.

The initial steps in M3’s procedure were largely the same as M1 and M2. Laboratory processing involved the measurements of three points on the cut surface from the fiducial reference. The third point was identified as the furthest away material from the bisection surface, the crest, of the bone dissection’s surface.

The fundamental computational input difference of M3 against M1 and M2 are that three splines were marked up instead of two. Where the third spline, $S_{3}$, followed the crest of the bone.

**Fig. 3:**
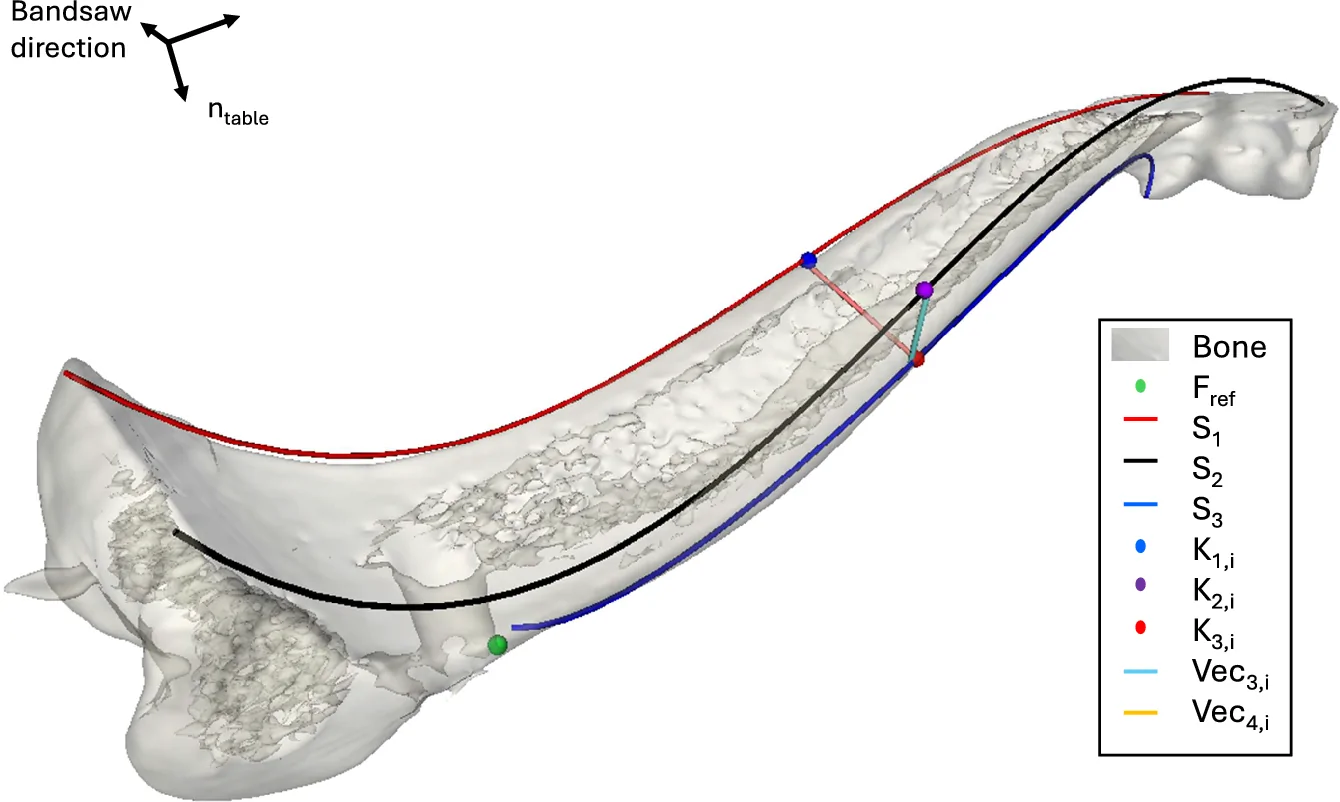
$\boldsymbol{Vec_{3}}$ and $\boldsymbol{Vec_{4}}$ displayed on the two mark-up splines, 3D Python diagram render using trimesh

All splines were modelled in *Python*, with three intersections identified using the same manipulations as Step 2. These intersection points enabled calculation of $\boldsymbol{Vec_{3}}$ and $\boldsymbol{Vec_{4}}$ through point subtraction. The cross product of these vectors determined $N_{3}$, the vector normal to the cut surface, which was subsequently used in a dot product to derive the plane equation. This approach constrained the plane assignment in 3D space, eliminating the need for bisection table cut assumptions (Fig. 3).

### Validation procedure

#### Slab width error

**Fig. 4:**
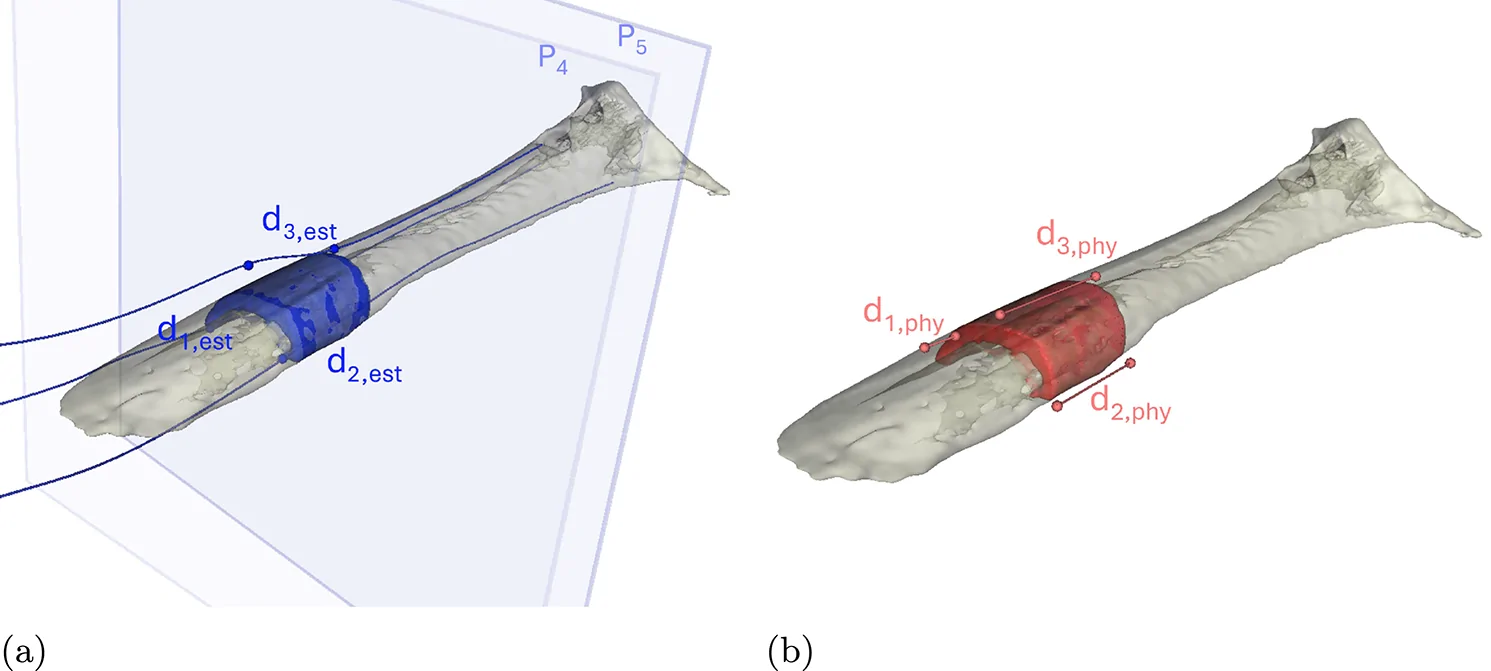
Comparative Euclidean distances, from Phillips et al. (2025) Fig. 3, under Creative Commons license [11]. Euclidean distances ($d_{1,est}$, $d_{2,est}$ and $d_{3,est}$) from computations were compared to physical measurements ($d_{1,phy}$, $d_{2,phy}$ and $d_{3,phy}$) for evaluating plane assignment. Image generated using* 3D Slicer*. **a** Computed dissection (blue), between two **b** Visualising dissection (red) as handled in subsequent planes (P4 and P5), overlaid on the laboratory the bone model.

After each dissection, validation measurements ($d_{phys}$) were taken between three intrinsic landmarks: two at the bone bisection edge intersections and one following the bone crest on the cut face. Euclidean distances between $K_{X,i}$ and $K_{X,i+1}$ points were used to calculate $d_{est}$ for comparison with these. This validation method was identical to that used in previous work [11] (see Fig. 4).

Measurements $d_{1,phys}$ and $d_{2,phys}$ on the bisection surface edges, were primarily used to evaluate the precision and accuracy of $d_{1,est}$ and $d_{2,est}$ computations, while $d_{3}$ measurements following the dissection crest compared performance between the three methods that define this third degree of freedom differently.

### Conceptual basis

#### Method 2

The vector $\boldsymbol{Vec_{2}}$ used to form planar equations in M2 differ from M1 while $\boldsymbol{Vec_{1}}$ remained the same. These vectors were chosen, primarily, to handle the concave shaped bones that were potentially overlooked in M1.

$\boldsymbol{Vec_{2}}$ was between the endpoint of a markup spline to the intersection point of the previous cut. During a specimen cut up, a concave bone rests on its endpoints on one side, and on the intersection points of the most recent cut. The cut plane’s pitch is thus perpendicular to this vector, which agrees with the orientation of the band saw.

#### Method 3

The third spline intends to lock a rotational degree of freedom of the calculated cut planes, preventing assumptions on vector directions. This is done by introducing two vectors that are known to be on the cut plane surface, since $S_{3}$ introduces a third point on the plane. The cross product of these two vectors describes the plane.

## Results

All methods had the same $d_1$ and $d_2$ error as the calculation was identical between methods (see Table 1). M3 had the lowest $d_3$ error which represents the error along the bone crest.

**Table 1:** Mean error in computed dissection measurements across 38 dissections, 7 bisections (with standard deviations [SD] in parentheses)

	Errors $d_1$ & $d_2$ $\,{\mathrm{mm}}$	Error $d_3\,{\mathrm{mm}}$
M1	*0.18 (1.5)* & *0.19 (1.5)*	*0.12 (1.7)*
M2	*0.18 (1.5)* & *0.19 (1.5)*	*0.06 (1.9)*
M3	*0.18 (1.5)* & *0.19 (1.5)*	*0.02 (1.5)*

A Friedman test was conducted to compare the median $d_{3}$ errors across the three methods (M1, M2, and M3). The analysis yielded a p-value of 0.058, indicating that there was no statistically significant difference in the accuracy of the 2D plane localization between the three methods at the standard *α = 0.05* level. These results suggest that the methods perform with comparable geometric precision, despite the differing underlying assumptions regarding specimen orientation.

**Fig. 5:**
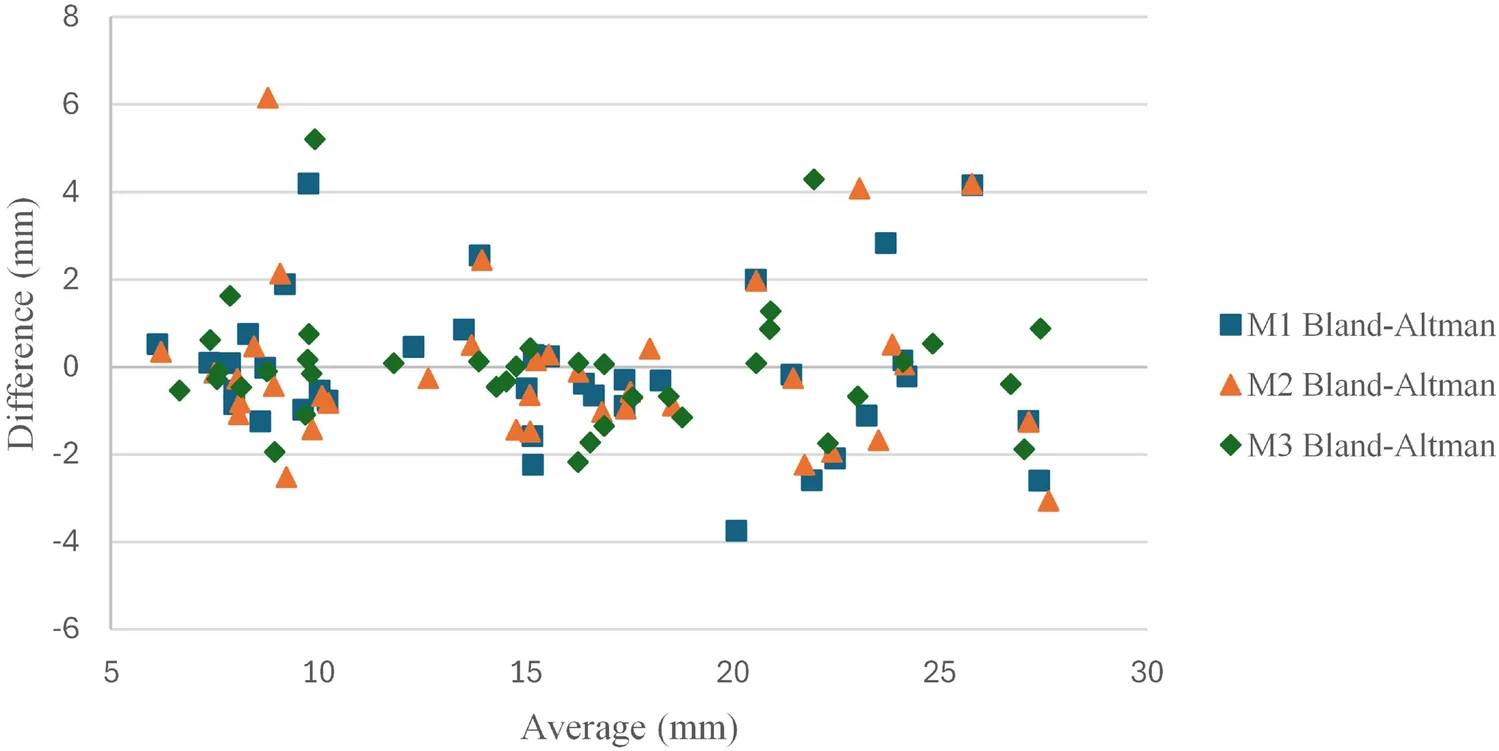
Plot of $d_3$ errors using original splines from M1, M2 and M3. 95% confidence intervals: M1 $\pm \,3.3\,{\mathrm{mm}}$, M2 $\pm \,3.7\,{\mathrm{mm}}$, M3 $\pm \,2.8\,{\mathrm{mm}}$

In Fig. 5 the difference in slab width measurements ($d_{est} - d_{phys}$) is plotted on the Y-Axis while the average between these $({d_{est} - d_{phys})}/2$ is plotted on the X-axis.

When an experienced *3D Slicer* user familiar with the cut-up procedure reinterpreted markup splines at the extremes of plausible placement $d_{3}$ errors increased to: M1 $0.15\pm 3.4\,{\mathrm{mm}}$, M2 $0.10\pm 4.0\,{\mathrm{mm}}$, and M3 $0.06\pm 2.8\,{\mathrm{mm}}$.

## Discussion

Two novel methods (M2 and M3) enable the orientation of 2D image planes within 3D volumes. These methods can facilitate the co-registration of histology with radiology following concave and more freeform bone bisection.

M1, M2, and M3 produced similar magnitudes of $d_{3}$ errors. Testing on specimens confirmed consistently small errors across expanded geometric transformations from a previous co-registration framework [11]. These errors were of consistent magnitude and small relative to current resection margins. Geometric transformation error evaluation will support 3D histology co-registration. It will contribute to more objective pre-surgical segmentation error predictions.

The calculation of intersections $K_{1,i}$ and $K_{2,i}$ was identical between all methods. Differences of error between $d_{1}$ or $d_{2}$ were small relative to the standard deviation (SD). Mean $d_{3}$ errors exhibited differences between methods and were also small relative to SD. The increase in $d_{1}$ and $d_{2}$ error against any $d_{3}$ was attributed to random variation - supported by the random scattering around 0 and absence of funneling which indicates difference are consistent across magnitudes (refer to Fig. 5). All M1, M2, and M3 confidence intervals overlap zero.

While the standard deviations for M2 and M3 remain higher than their respective mean errors, these methods offer greater procedural robustness in a clinical workflow. Unlike M1, which requires a specific orientation of the bone on the bandsaw table to satisfy perpendicularity assumptions, M3 is independent of the physical resting position. By utilizing a third measurement to constrain the plane in 3D space, M3 accommodates complex, non-convex geometries where stable physical orientation is difficult to achieve. Given that current surgical wide margins are typically 10–20 mm, a standard deviation of < 2 mm represents a clinically acceptable level of variance that could contribute to relatively more accurate evaluation compared to the clinically accepted wide surgical margins.

Statistical analysis using a Friedman test yielded a p-value of 0.058. While this does not meet the traditional threshold for statistical significance (*p* < 0.05), it suggests that the three methods perform with comparable accuracy. The lack of a significant difference between the established M1 and the novel M3 is noteworthy; it indicates that M3 achieves similar geometric precision without requiring the restrictive geometric assumptions or specific specimen orientations necessitated by M1. Consequently, M3 can be utilised for complex bone geometries without sacrificing the accuracy established in previous frameworks.

Long bone geometry was tested using canine specimens, though further testing with more complex geometries, such as the pelvis, is recommended. M3 is expected to be effective for these more freeform anatomical structures where initial bisection can be of particular challenge. M3 constrains planes in three dimensions and reduces reliance on quality and classification of the bisection surface.

Established laboratory dissection protocols and secured CT scanner access enabled total data acquisition in under one hour per specimen. Further computational processing was executed with negligible latency. Implementation of M1, M2, or M3 within a histology co-registration framework could result in minimal disruption to clinical workflows. Limited initial macroscopic histology assessment may also be attained on 2D bisections before CT scanning. The entire process consistently required less than one hour and minimal manual input for each specimen, comparing favorably to the up to 8-hour processing times reported for other tissue [24].

Micro-computed tomography (microCT) presents an enhancement to this pipeline due to its isotropic voxel sizes of approximately $$10\,\upmu {\mathrm{m}}$$$$$$$$$$$$$$$$$$$$$$$$$$$$$$$$ $$ [29] compared to conventional CT’s resolution of $$250\,\upmu {\mathrm{m}} \times 250\,\upmu {\mathrm{m}} \times 500\,\upmu {\mathrm{m}}$$. Histology provides higher in-plane resolution (approximately $$5\,\upmu {\mathrm{m}} \times 5\,\upmu {\mathrm{m}}$$) [23]. However, microCT does not replace histology, as it cannot resolve cellular-level pathological features, and remains relatively costly and uncommon in routine clinical workflows [10, 30].

Several limitations affect the results. The use of vernier callipers proved difficult due to the complex geometry around bone condyles and the presence of incompletely debrided soft tissue. Some bisections were also more freeform, meaning they rested on three distinct points or less during axial bandsawing, rather than four. This impacted the vector assumptions made in M1 and M2 which exhibited slightly larger SD than $d_{3}$ errors.

M3’s reliance on $S_{3}$ without a clear reference point introduced potential analytical error. When markup splines were reinterpreted at their extreme plausible positions, M3’s error increased to $0.06\,{\mathrm{mm}}$, with the SD rising from $1.5\,{\mathrm{mm}}$ to $2.8\,{\mathrm{mm}}$. Despite this increased variability, the similar error magnitude indicates a level of robustness in light of surgical margins used clinically. M3 is conceptually most suitable for highly freeform bisection cuts, such as those in the ilium.

Our work replicates findings from previous studies using a larger specimen cohort whilst proposing two additional geometric transformation methods.

### Opportunities for further development

The development of M1 was a proof-of-concept initiative in pervious work, with M2 and M3 providing alternative calculations for addressing challenges assigning planes in bone sarcoma histology co-registration. However, there remain significant opportunities for further refinement and advancement:

*Expanded testing* Further evaluation with a greater number of specimens is essential. Consideration must be given to more concave and freeform bones structures with misaligned endpoints.

*Lowest error prioritisation* Plane orientations with the lowest slab width error should be prioritised for diagnostic information. Clinical measurement of $d_{phy}$ alongside measurement from $F_{ref}$ to $K_{1{ \& }2{ \& }3,i}$ may enable co-registration optimisation by selecting the best geometric transformation method for different bone geometries within the same specimen. However, this will introduce more solution complexity and needs investigation.

*Development of a 3D Slicer module* Creating an in-house *3D Slicer* module would streamline the process by eliminating the need to import/export.json files and execute multiple Python scripts. This integration could further reduce processing times and simplify the histology co-registration workflow into a single, more user-friendly interface.

*Co-registration validation* The Dice Similarity Coefficient, which ranges from 0 (no overlap) to 1 (complete overlap), could quantify co-registration accuracy by comparing histology bone labels with radiology segmentation. This would be valuable for comparing the performance of bone sarcoma co-registration against that in other tissues.

### Clinical procedure implications

The integration of clinically viable bone sarcoma histology co-registration will provide minimal disruption to regular treatment workflows [13]. Future patient information should include pre-surgical radiology data to compare against ex-vivo CT and histology images. A comprehensive data bank from this would inform decisions on surgical margins and treatment pathways [31]. With wider adoption, the accumulation of accessible data will grow. In turn, providing surgeons with a robust foundation for improved decision-making, ultimately enhancing patient outcomes.

To maximise accuracy, clear technical protocols are essential. When slicing concave bone specimens, the bone should consistently rest on four corner points to align with M2’s underlying principles. Where this is not feasible due to freeform geometries, a third reference point should be identified and measured for each bisection — in our study, this was the point furthest from the bisection surface.

## Conclusion

This study presents a development on previous methods for the co-registration of 2D histology images into 3D radiology volumes. The proposed computations calculate plane orientations, utilising multiple reference points and vectors to do so. It leverages existing, open-source, and widely-accessible tools such as *Python* and *3D Slicer*.

The results from error analysis show the methods M2 and M3 are robust and suitable for plane location identification. Of 38 dissections across 4 specimens, $d_{1}$ and $d_{2}$ yielded errors of $0.18\,(1.5)\,{\mathrm{mm}}$ and $0.19\,(1.5)\,{\mathrm{mm}}$ respectively. Regarding $d_{3}$, M1 produced an error of $0.12\,(1.7)\,{\mathrm{mm}}$, M2 $0.06\,(1.9)\,{\mathrm{mm}}$, while M3 $0.02\,(1.5)\,{\mathrm{mm}}$.

## Data Availability

The data collected from specimen processing is included in the GitHub repository.

## Code availability

https://github.com/phiro753/Py-OrthoRadPathFusion
